# Rayleigh wave attenuation and phase velocity maps of the greater Alpine region from ambient noise

**DOI:** 10.1038/s41598-024-80729-z

**Published:** 2024-11-25

**Authors:** Henrique Berger Roisenberg, Fabrizio Magrini, Irene Molinari, Lapo Boschi, Fabio Cammarano

**Affiliations:** 1 Dipartimento di Scienze, Università degli Studi Roma Tre, Rome, Italy; 2 Research School of Earth Sciences, The Australian National University, Canberra, Australia; 3 Istituto Nazionale di Geofisica e Vulcanologia, Sezione di Bologna, Bologna, Italy; 4 Dipartimento di Geoscienze, Università degli Studi di Padova, Padua, Italy

**Keywords:** Rayleigh wave attenuation, Seismic tomography, European crust, Solid Earth sciences, Geology, Geophysics

## Abstract

We use seismic ambient noise data from 724 publicly available broadband seismic stations across central Europe to create detailed phase velocity and attenuation maps of Rayleigh waves, focusing on short periods down to 3 s. We interpret these maps in terms of the underlying physical processes relevant to the nature of continental crust. Through a regionalized interpretation based on tectonic settings, we highlight the significant role of fluid-filled fractures in the attenuation of surface waves. Our findings indicate a close connection between the time elapsed since the last tectonic activity in the European crust and the attenuation coefficient values. Additionally, we observe a pronounced decrease in attenuation coefficient values at periods below 6 s. The anti-correlation between attenuation coefficient and phase velocity in recently active tectonic regions suggests that fluid-filled fractures are likely the dominant factor governing seismic attenuation in the European crust.

## Introduction

**Fig. 1:**
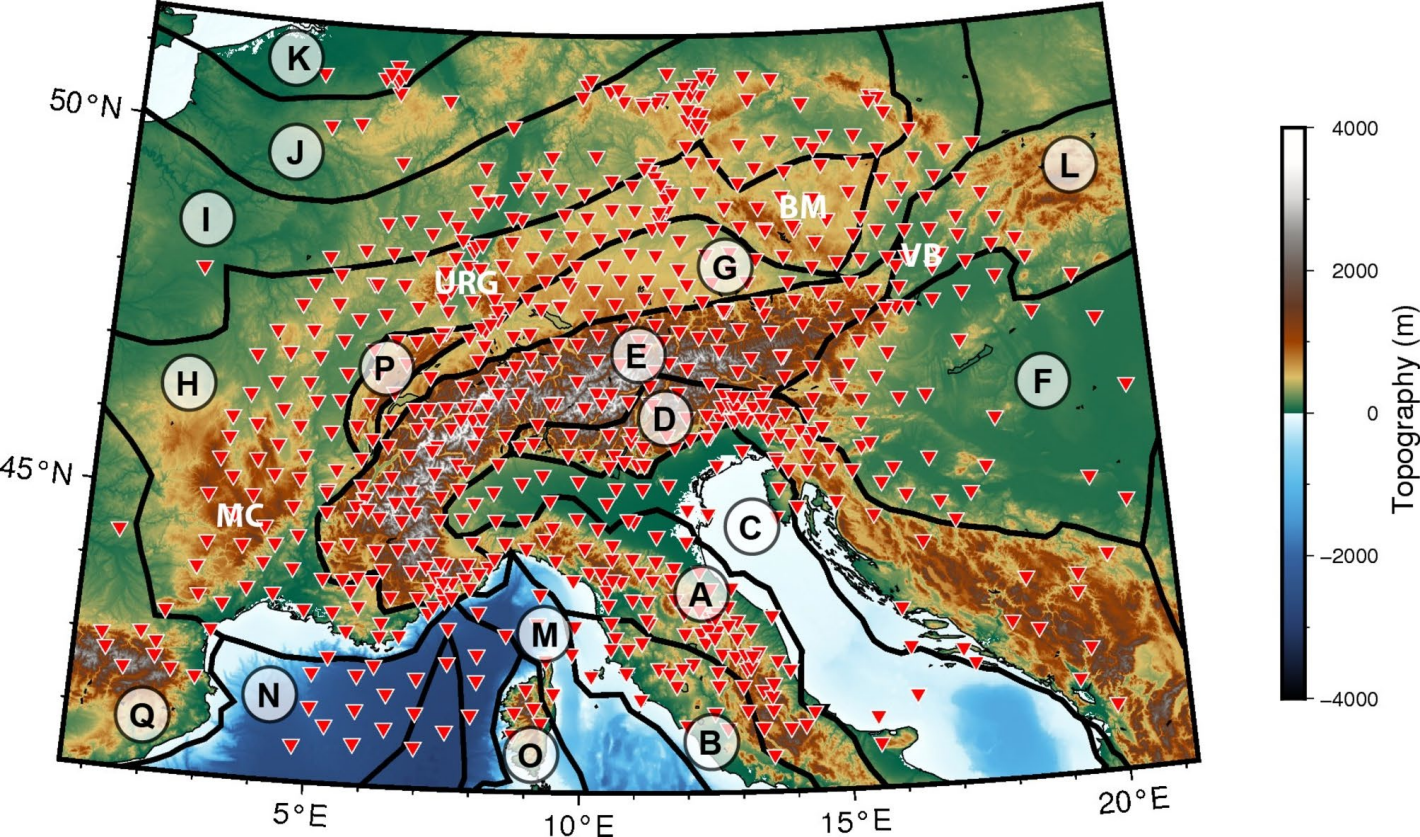
Topographic map of the greater Alpine region. Black lines delimit the main geotectonic provinces of the area based on the recent compilation by Hasterok et al. (2022)^43^: (A) Apennine orogen, (B) Campanian arc, (C) Adriatic-Apulian Foreland, (D) Dinarides, (E) Alps, (F) Pannonian basin, (G) Molasse basin, (H) Moldanubian belt, (I) Saxo-Thuringian belt, (J) Rheno-Hercynian belt, (K) Avalonia, (L) Carpathian mountains, (M) Tyrrhenian basin, (N) Balearic basin, (O) Sardinia-Corsica block, (P) Jura mountains, (Q) Cantabrian-Pyrenean belt. Red triangles denote the locations of seismic stations. MC: Massif Central, URG: Upper Rhine Graben, BM: Bohemian Massif, VB: Vienna Basin. This map was created using PyGMT^44^.

The European continental crust has undergone a complex evolution, shaped by significant tectonic events. The central European crust, as we know it today, was predominantly formed during three main orogenic episodes: the Variscan, Alpine, and Apennine orogenies (Fig. 1). Originating in the Paleozoic era, the Variscan or Hercynian orogeny resulted from multiple collisional stages that culminated in the assembly of Pangea^1,2^. During the Late Mesozoic, the Alpine belt emerged due to the subduction of the Tethyan oceanic crust following the breakup of Pangea, coupled with the collision between the African and Eurasian plates^3^. Subsequently, in the Cenozoic era, the Apennine belt formed through the westward subduction of the Adriatic plate, with its peak activity occurring from the Miocene to the Pleistocene epochs^4^. It was also during the Cenozoic that the Variscan domain underwent significant post-orogenic events, such as the formation of the European Cenozoic Rift System (ECRS) and the intraplate vulcanism.

These orogenic systems, specially the Alpine orogeny, have been the focus of many studies employing various geophysical methods to understand their complexity and heterogeneity^5–7^. Numerous deep reflection/refraction seismic surveys^8–10^, receiver function analyses^11–13^, gravimetric^14,15^ and local earthquake tomography studies^16,17^, and, more recently, ambient-noise tomography^18–27^ have contributed to unveiling the geological and tectonic characteristics of this orogenic system.

Despite these advancements, a knowledge gap remains regarding the dominant mechanisms governing seismic attenuation in the European crust. Seismic attenuation carries important information that, when combined with seismic velocity, holds the potential to unravel the thermo-chemical and rheological conditions of the subsurface^28–32^. This is crucial for addressing ambiguities related to the temperature, density, and viscosity of the crust^32^.

The AlpArray program^33^ was developed to apply multiple geophysical investigation methods to study the European crust, more specifically the greater Alpine region. To reach its goal, the first step has been the implementation of a dense seismological network of broadband sensors, similar to USArray^34^ and IberArray^35^, capable of imaging lithospheric and upper mantle structures at unprecedented resolution^33^. This initiative provides us with a large amount of data that can be used to study seismic attenuation.

To address the existing gap in seismic attenuation measurements in the European crust, in this work we apply a novel method^36–38^ to estimate Rayleigh wave attenuation ($\alpha$) from seismic ambient noise in the greater Alpine region (Fig. 1), covering the three main orogenic belts. The waveforms were processed using a robust cross-correlation procedure, successfully tested, and previously applied at different tectonic settings^26,32,36–42^. This allows us to analyze relatively high frequencies compared to earthquake-based surface wave tomography and can be applied even in tectonically stable regions, where earthquakes are scarce. Two-year-long recordings from 724 publicly available broadband seismic stations were processed and analyzed, resulting in the first-ever Rayleigh wave attenuation maps covering the entire Alpine region. These were jointly interpreted with our high-resolution Rayleigh wave phase velocity maps.

## Results

### Phase velocity

**Fig. 2:**
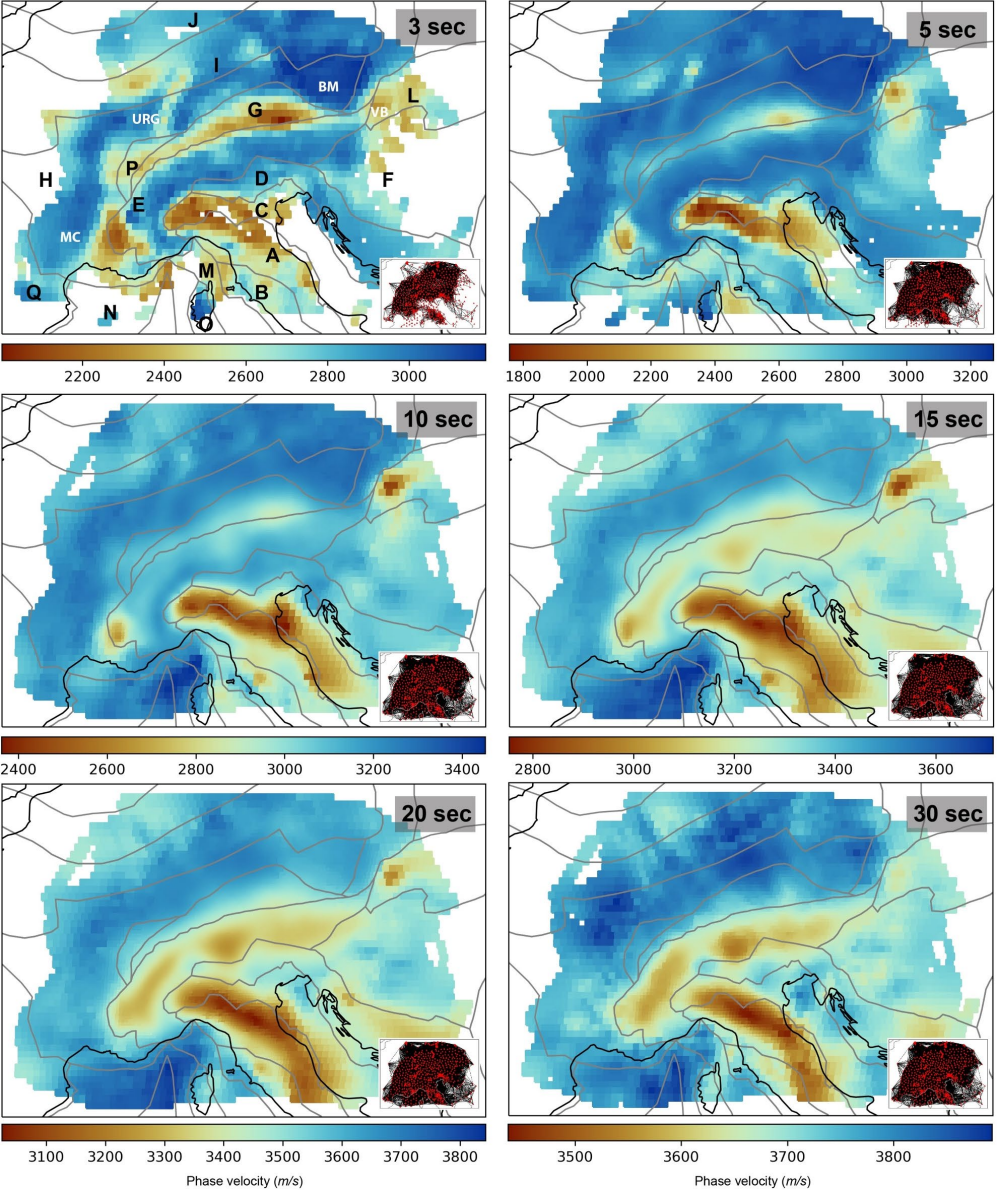
Rayleigh wave phase velocity maps at 3, 5, 10, 15, 20, and 30 s. For the geotectonic provinces refer to Fig. 1. Lower right panels show raypath maps at each period. Red triangles represent the broadband seismic stations.

Due to the dense data coverage in our study area, phase velocity maps were created using an adaptive grid based on the number of raypaths crossing each pixel (Fig. 2). Details about the calculation and plotting of the phase velocity maps are available in the “Data and methods” section.

Our Rayleigh wave phase velocity maps (Fig. 2) correlate well with the geology of the study area. Particularly at 3 s, the main geological regions are easily identified. At this period, the Adriatic-Apulian Foreland, the Molasse basin, the Apennines, the Campanian arc, the Thyrrhenian basin, and the contact between the Eastern Alps and Western Carpathians, where the Vienna basin is located, are all characterized by low velocities, from 2.1 to 2.6 km/s, while the Alps, the Dinarides, the Moldanubian, the Saxo-Thuringian, and the Rheno-Hercynian belts are characterized by relatively high velocities, from 2.8 to 3.2 km/s (Fig. 2). Even smaller structures, related to the ECRS, are highlighted as low-velocity zones in the 3 s map, the most apparent one being the Upper Rhine Graben.

At 5 s the Molasse basin becomes much less evident, while the Adriatic-Apulian Foreland is strongly highlighted by its low velocity (Fig. 2). As the period increases, the Adriatic-Apulian Foreland, along with the Apennines, emerge as the primary zones of low velocity. By 15 s, the Alps, the Dinarides, and the Carpathians also exhibit relatively lower velocities compared to other tectonic settings (Fig. 2).

Rayleigh wave velocities across all periods consistently show higher values in the Moldanubian, Saxo-Thuringian, and Rheno-Hercynian belts compared to other tectonic regions (Fig. 2). Similarly, velocities in the Tyrrhenian and Balearic basins are above average for periods longer than 10 s (Fig. 2). Among the major sedimentary basins in Europe, the Pannonian basin consistently exhibits the highest velocities across all periods.

**Fig. 3:**
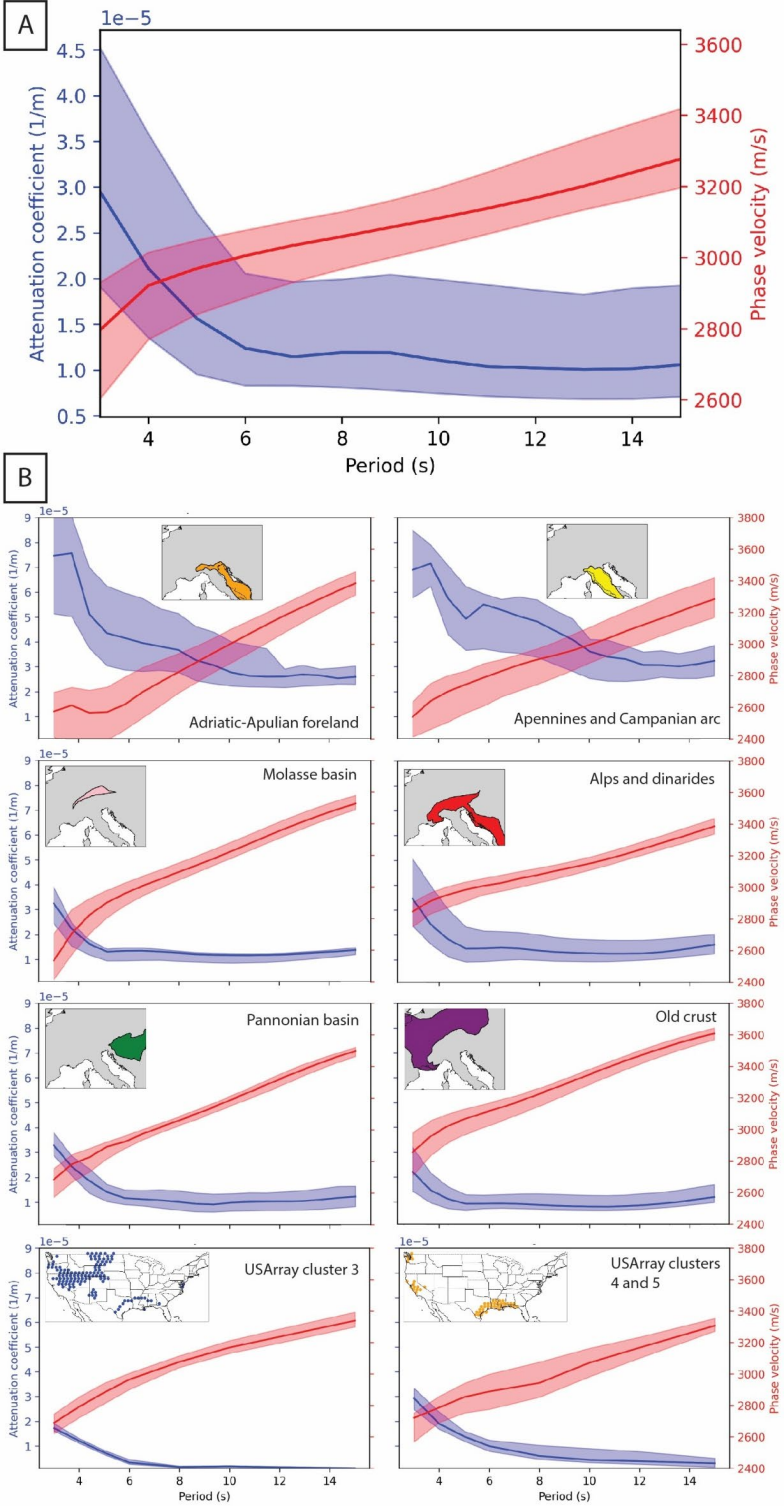
(**A**) Variation of median phase velocity and attenuation coefficient with period for the whole study region. The upper and lower bounds represent the percentiles 75 and 25, respectively. (**B**) Regionalized analysis of attenuation coefficient and phase velocity values. The absolute values of phase velocity change, but always follow the same increasing pattern, while the attenuation coefficients are different depending on the tectonic setting. USArray clusters 3, 4, and 5^32^ are shown for comparison.

As expected, the median value of Rayleigh wave phase velocity grows with increasing period, ranging between 2.75 and 3.25 km/s (Fig. 3A). At longer periods (15–30 s), the low-velocity zones are related to the portions of the crust with deeper crustal roots, i.e., the Alps and Apennines, while the highest velocities are seen in the region of the Balearic and Tyrrhenian basin, where the Moho is shallower.

### Attenuation maps

**Fig. 4:**
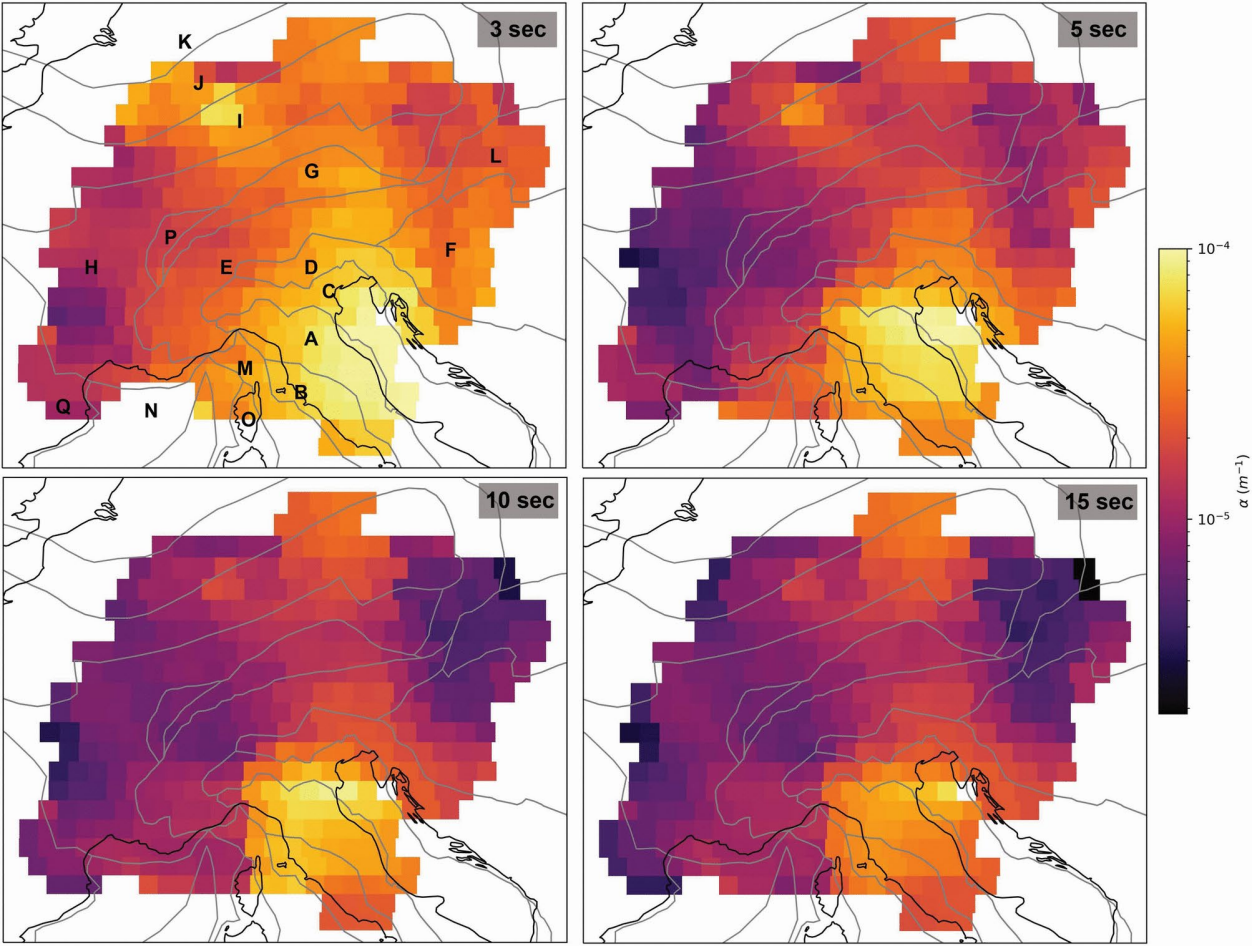
Rayleigh wave attenuation coefficient maps for 3, 5, 10, and 15 s. For the geotectonic provinces refer to Fig. 1.

In comparison with the phase velocity, the correlation of the attenuation maps with geology is less clear. Attenuation coefficient values are the highest at low periods (Fig. 4), especially in the Adriatic-Apulian Foreland and in the Apennines at 3 and 5 s (Fig. 4). A general pattern, with high attenuation in the southeast and low attenuation in the west, is found at 3 s. From 5 s, this pattern extends, with low $\alpha$ also in the eastern portion of our maps (Fig. 4).

At 10 and 15 s, the westernmost Moldanubian and Saxo-Thuringian belts are characterized by relatively low $\alpha$ (Fig. 4). These regions, together with the NE portion of our map, comprising the western Pannonian basin, the western Carpathian Mountains, and the eastern Rheno-Hercynian belt, are the lowest-attenuating regions of the study area (Fig. 4).

Figure 3A shows that the attenuation coefficient decreases with increasing period in a non-uniform way, i.e., much more steeply between 3 and 6 s than beyond 6 s. On the other hand, the phase velocity values grow with increasing period, presenting a larger growth from 3 to 6 s and then linearly increasing.

## Discussion

In a nutshell, our results can be summarized as follows: (1) at short periods the Rayleigh wave phase velocity maps correlate with known geological features (Fig. 2); (2) average phase velocity grows linearly with period at periods larger than 6 s (Fig. 3A); (3) the Rayleigh wave attenuation coefficient is high in sedimentary and tectonically active areas (Fig. 3B and 4); (4) the rate at which the attenuation coefficient decreases with period changes at least once with changing period (Fig. 3).

Earlier surface-wave tomography studies of the Alpine area are very similar to ours^18,20,22^. The advantage of our maps is that we calculated for the first time velocities down to 3 s, and thanks to the large number of stations we achieved a much finer lateral resolution.

Before attempting to interpret our results, it is important to quantify their resolution. Our station coverage is almost three times denser than that of a previous study using the same methodology in the contiguous United States^32^. The phase velocity map at 3 s relies on average on 33 raypaths per pixel, with some pixels sampled by up to 140 raypaths (Fig. 2). Figure S1 presents the resolution test performed for our maps at 3, 5, 10, and 30 s, showing where the lateral resolution is relatively high. This allowed us to parametrize attenuation maps with $0.5^{\circ }$ equal-area cells, which we consider appropriate for the geological heterogeneity of the region. The comparison of our new information with the jointly obtained phase velocity maps can therefore shed light on the physical properties of the crust.

### The role of temperature and fractures

Intrinsic anelastic attenuation in the Earth’s crust is known to depend on temperature, water content, and partial melting^31^. Global heat flux maps^45^ show that Central and South Italy, the Western Mediterranean, the Pannonian basin, and central France are characterized by high surface heat flux, while the Adriatic, the Po basin, and Eastern Mediterranean are characterized by low surface heat flux. These regional variations of temperature are not correlated with our maps of $\alpha$. For example, the Adriatic and Po basins are characterized by low surface heat flux^45^, and relatively high attenuation, while the region of the French Massif Central has low attenuation, despite its high surface heat flux^45^, following the opposite pattern than what would be expected if temperature was the main cause of $\alpha$ heterogeneity. This is in agreement with what was observed in the conterminous United States of America^32^. These observations corroborate the idea that temperature alone does not control the attenuation of surface waves in the upper crust^46^.

Previous studies^32,46,47^ advocate the importance of fluid-filled fractures on the absorption of seismic waves. To understand how these fractures can influence the attenuation coefficient values in our study area, we analyze $\alpha$ heterogeneities laterally, comparing their pattern with the age and lithology of rocks.

The study region has gone through three different orogenic stages: the Hercynian, the Alpine, and the Apennine orogenies. The first one was responsible for the formation of the Moldanubian, the Saxo-Thuringian, and the Rheno-Hercynian belts. Taken together, these provinces form what we have dubbed “old crust”. The attenuation coefficient values vary little in these regions and are the lowest, while the phase velocity values are the highest found in our study region (Fig. 3B). The largest $\alpha$ values and lowest phase velocities of this area are located in the ECRS, specially at 3 s (Figs. 2 and 4).

The Late Mesozoic Alpine orogeny was responsible for the formation of the Alps and the Dinarides, which are slower and more attenuating than the old crust. In addition, $\alpha$ is more heterogeneous there than in the old crust. A sharp decrease in attenuation coefficient values occurs up to 6 s. Above this period, $\alpha$ is approximately constant (Fig. 3B).

In the Adriatic-Apulian Foreland and the Apennines, formed during the Apennine orogeny in the Cenozoic, $\alpha$ changes most rapidly with period, at all periods (Fig. 3). The rate of decrease of $\alpha$ is larger at smaller periods, and the attenuation coefficient and phase velocity are anti-correlated. On the other hand, the Molasse and Pannonian basins, although closer in age to the Adriatic-Apulian Foreland, present similar $\alpha$ and phase velocity variation to the Alps and Dinarides. In general, we see that the absolute value of phase velocity changes, but always follows the same increasing trend with period, while the rate of change of the attenuation coefficient depends on the tectonic setting.

Fluid-filled fractures and pores can cause an increase in the absorption of seismic waves while at the same time reducing seismic wave velocities by reducing the shear modulus of the rocks, resulting in attenuation coefficient and phase velocity being anti-correlated. This is most apparent in the tectonically-active recent crust. In general, the time elapsed since the last tectonic activity and the intrinsic attenuation is anti-correlated^46^. Based on geology, we expect the recent crust to have more fractures and pores filled with fluids than older, more stable regions. With increasing depth, i.e., with increasing period, fractures and pores close, and $\alpha$ becomes less heterogeneous. The fluids on the cracks can also be lost to retrograde metamorphism^46^, reducing the values of $\alpha$. This could explain the different attenuation patterns according to period and the change in attenuation-vs-period gradient at around 6 s. We infer that most of the attenuation heterogeneity measured in the study area could be related to fluid-filled fractures.

The same data processing method has already been applied to USArray data^32^, with qualitatively similar results. In particular, anti-correlation between $\alpha$ and phase velocity was found to be maximum at around 4 s. In the contiguous United States, a sharp decrease in $\alpha$ is found at 6–8 s. The attenuation and phase velocity values of previously published clusters 3, 4, and 5 for the United States^32^ are comparable to those found in this study. Cluster 3, which partially corresponds to the Rocky Mountains and the Intermontane plateaus, is similar to the European old crust (Fig. 3B). Clusters 4 and 5^32^, comprising the coastal plain and westernmost US sedimentary basins, are similar to the Molasse and Pannonian basins (Fig. 3B).

The correlation between high attenuation and low velocity, which is linked to high fault and fracture density, as well as fluid-filled sediments, has already been observed in the Southern Alps region^48^. Regions of high attenuation in the brittle crust are often linked with frequent microtremors and faults^49^. This is further reinforced by studies on Apennines earthquakes^50–52^. The AlpArray seismic catalogue^53^ reveals that low-magnitude events (2.4–3.5 Mw) are more prevalent in the Po Plain and Apennines compared to other regions covered by the experiment. Additionally, within the first 4 km depth, the occurrence of low-magnitude events is visibly higher in these regions compared to others. These findings, combined with the observation of a larger attenuation coefficient in the same region, lend further support to the hypothesis that fluid-filled cracks and fractures control surface wave attenuation.

### Limitations of our model

Attenuation tomography is challenging in several ways, which is why studies of this type are less common and consistent compared to velocity tomography, which has been widely used over the past 40 years^54^. Among the general challenges reported in the literature are the effects of focusing and defocusing, scattering, site amplification, noise directionality, and slowness inhomogeneity^55^. Estimates of surface wave attenuation might also be affected by the presence of body-wave signal not accounted for by the theory and by differences in the terrains where the data were collected^40^. Another issue is that seismometers are often poorly calibrated to amplitude measurements^56^.

**Fig. 5:**
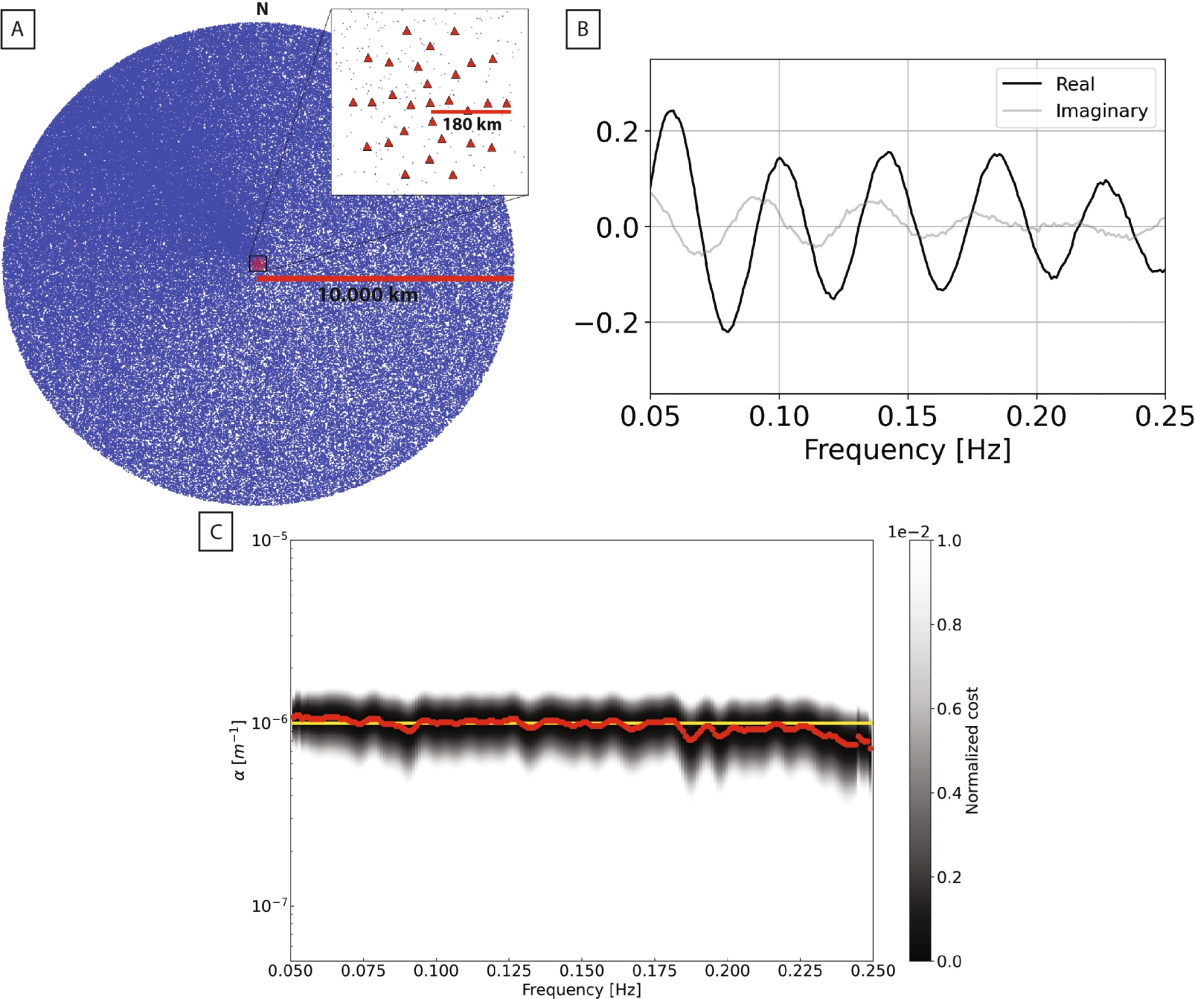
(**A**) 200,000 points sources distributed with a higher density far from the centre of the array, and to the Northwest. The receivers are uniformly distributed with a radius of 180 km. (**B**) Real (black) and imaginary (gray) parts of synthetic data cross-correlation at two stations from the virtual array in A, with interstation distance of 67,600 m, after ensemble-averaging over 25,000 realizations. (**C**) Cost function $C(\alpha ,\omega )$ associated with our synthetic test, shown as a function of attenuation coefficient and frequency. Red dots mark the values of $\alpha$ for which $C(\alpha ,\omega )$ is minimized at each frequency; the yellow line indicates the assumed attenuation model $\alpha = 1\times 10^{-6}$ m^–1^, used to generate the synthetic data.

The inversion technique applied in this and previous studies^32,36–38,40,57^ differs from alternative methods^30,58^, to increase the feasibility of the attenuation retrieval and its interpretation^38,40,55^. A challenge related to measuring surface wave attenuation in the European crust is the nonuniformity of source distribution^58–60^. To quantify its effects, we performed a synthetic test using 200,000 point sources, nonuniformly distributed, with a higher density of sources to the Northwest and far from the centre of the array (Fig. 5A). This accounts for the fact that most seismic noise recorded in Europe is associated with microseisms in the North Atlantic. After combining the results of 25,000 realizations^38^, we verified visually that synthetic cross-correlations were converging to the theoretical Green’s function (Fig. 5B). The “input” model has uniform $\alpha = 1\times 10^{-6}$ m^–1^ and uniform Rayleigh wave velocity at each period. Next, the cost function (Eq. 1) was evaluated via a 1-D grid search over 350 values of $\alpha$ evenly spaced on a logarithmic scale between 5 $\times$ $10^{-8}$ and 1 $\times$ $10^{-4}$ m^–1^. The cost-function minima correspond quite accurately to the theoretical value of $\alpha$ (Fig. 5C). The results of this test show that surface wave attenuation can be estimated based on ambient noise, even when the noise field is not exactly diffuse.

Another test that we performed (Fig. S2) consisted of selecting a 39-station array located in a complex geological region, between the Adriatic Sea, the Alps, the Dinarides, and the Po Plain. By randomly eliminating stations at different azimuths inside this subarray, and recomputing $\alpha$, we verified that the change of amount and distribution of stations inside a subarray does not affect significantly the inverted attenuation coefficient values.

In our approach, based on ambient noise cross-correlation, the effect of focusing and defocusing is probably negligible, since the receivers that we correlate are only a few wavelengths away from each other. Other benefits of the method are: that phase velocity is determined by a preliminary independent inversion, and is a fixed parameter of the attenuation inversion, which is preferable to constraining attenuation and velocity simultaneously^39,40^; that data is not averaged over distance nor azimuth, and the sum of misfits associated with each interstation pair is minimized, contributing to regularizing the inversion^55^; that Rayleigh wave overtone signal is removed before inversion, via time-domain processing of the cross-correlations^38^.

A possibly important limitation resides in the effects that incoherent noise has on the cross-correlation of ambient signal. Its effect on $\alpha$ estimates is difficult to predict, and it is possible, in principle, that it could result in $\alpha$ being overestimated. This will be the topic of further, methodological work.

## Conclusions

We used ambient noise cross-correlations to estimate laterally varying Rayleigh wave phase velocity, and, for the first time, attenuation across the greater Alpine region. We jointly interpreted our velocity and attenuation maps in terms of the nature of the European crust. The main implication of our results is that surface wave attenuation is controlled by the presence of fluid-filled pores and fractures, rather than by temperature variations. Our findings are corroborated by the previous application of our method to USArray data, and by independent results based on earthquake (as opposed to ambient) seismograms.

## Data and methods

This study is based on publicly available, two-year-long continuous recordings of 724 broadband seismic stations, comprising the AlpArray Seismic Network^33^ and adjacent available stations in the greater Alpine region. The data, recorded from 2017 to 2019, were used to calculate the Rayleigh wave phase velocity and attenuation maps via SeisLib^57^, an open-source Python package for multiscale seismic imaging.

Before obtaining Rayleigh wave phase velocity and attenuation, the waveforms were demeaned, detrended, tapered (5%), bandpass filtered between 0.004 and 2.5 Hz and deconvolved with the instrument response to get displacement.

### Rayleigh wave phase velocity

Rayleigh wave dispersion curves from the vertical component of recorded noise were retrieved using an automated algorithm implemented on SeisLib^57^, based on the fact that the frequency spectrum of the ambient noise cross-correlation of two stations approximately coincides with a zeroth order Bessel function of the first kind^39^.

Pairs of receivers that recorded simultaneously for at least 6 months were used in this study, to reduce the seasonality variation of seismic noise. As a first step to retrieve the phase velocity, the algorithm divided the seismograms into one-hour-long time windows with 50% overlap and cross-correlated in the frequency domain. Data from receivers more than 300 km away from one another were not correlated.

The extraction of a phase velocity at the zero-crossings of the Bessel function was resolved by setting a reference curve^21^ and automatically picking the dispersion curve closer to this curve, starting at relatively low frequencies, and extending to larger frequencies. For details on the automatic picking refer to previous work on SeisLib^57^. In total, 46,041 dispersion curves were retrieved, and are available in an online repository (link on the supporting materials).

Finally, a least-squares inversion algorithm based on ray theory^57,61^ was applied to the dispersion curves, resulting in phase velocity maps at different periods. The study area was discretized in adaptive grids. Initially, the region was parameterized in equal-area cells of $0.2^{\circ }$. The grid was then further refined for a maximum of two times, at cells sampled by at least 150 station-station paths. The minimum cell size achieved was $0.05^{\circ }$. Before carrying out the inversion a roughness-damping regularization was applied based on the L-curve criterion. For the current tomographic inversion a roughness coefficient of $3 \times 10^{-3}$ was chosen. The mathematical details of our least-square inversion approach have been previously published^57,61^ and are not the scope of this paper.

### Rayleigh wave attenuation

The main challenges and limitations of the estimation of attenuation using surface waves from ambient noise are reported in the “Discussion” section. A detailed review of the mathematical methods and limitations is presented in previous works^40,55^.

While our phase velocity estimates are related to the phase of surface wave cross-correlations, estimates of Rayleigh wave attenuation ($\alpha$) are related to the cross-correlation’s amplitude. To measure attenuation from seismic ambient noise, we applied the procedure developed by Magrini & Boschi^38^ based on the theory of Boschi et al.^36,37^. We used the automated algorithm implemented in SeisLib to calculate our Rayleigh wave attenuation values. In practice, Rayleigh wave attenuation is found by minimizing the cost function,

1 $$\begin{aligned} C(\alpha , \omega )= \sum _{i, j} \Delta _{ij}^2 \left| {\text {env}}\left[ \Re \left\{ \frac{u({\textbf{x}}_i, \omega ) u^*({\textbf{x}}_j, \omega )}{\left\langle |u({\textbf{x}}, \omega )|^2\right\rangle _{{\textbf{x}}}}\right\} \right] -{\text {env}}\left[ J_0\left( \frac{\omega \Delta _{ij}}{c_{ij}(\omega )}\right) \textrm{e}^{-\alpha (\omega ) \Delta _{ij}}\right] \right| ^2 \end{aligned}$$

where the indexes point to pairs of receivers, *u* denotes the ambient-noise recording in the frequency domain, and $*$ complex conjugation.

The first step of the calculation of attenuation involves the subdivision of the study area into subarrays. In our case, we decided to adopt a grid with blocks of $1.5^{\circ }$ each, with 50% overlap in latitude and longitude and containing at least six stations. Continuous recordings at each station were subdivided into 6-hour segments, and simultaneous segments within each subarray were cross-correlated. Cross-correlations were normalized by the average power spectral density found in each time segment. This enables the association between cross-correlation amplitudes and Rayleigh wave attenuation by isolating the parameters related to the frequency content and the spatial distribution of the noise sources^32^. It also mitigates the effects of anomalous signals such as large or nearby earthquakes. We considered only the subarrays with 8 or more stations pairs with more than 30 days of simultaneous recording. After this, we were left with 142 subarrays.

The interstation phase velocities estimated previously were used to calculate the Bessel functions of the station’s pairs of each subarray. The envelopes of the cross-correlations and the Bessel functions are found and equation 1 is solved to retrieve one attenuation coefficient ($\alpha$) value per frequency. The minimum of $C(\alpha , \omega )$ is found among 300 values of $\alpha$ geometrically spaced between $5 \times 10^{-8}$ m^–1^ and $10^{-4}$ m^–1^ . The attenuation maps were parameterized in $0.5^{\circ }$ equal-area cells. An attenuation value was assigned only to those with a minimum of two available attenuation curves.

## Supplementary Information

Supplementary Information 1.

## Data Availability

Data and Python scripts are open access, and provided as a compacted file downloadable in Zenodo, registered under the DOI: 10.5281/zenodo.10839031.
