# Supplementary material for: Rayleigh wave attenuation and phase velocity maps of the greater Alpine region from ambient noise
Source: Sci Rep. 2024 Nov 25;14:29164. doi: 10.1038/s41598-024-80729-z (PMC11589122; doi:10.1038/s41598-024-80729-z)
Supplement: Supplementary file 1 — Supplementary Information 1. [file 41598_2024_80729_MOESM1_ESM.pdf]

Supporting Information: Rayleigh wave attenuation and phase velocity maps of the greater Alpine region from ambient noise

Henrique Berger Roisenberg[1], Fabrizio Magrini[2], Irene Molinari[3], Lapo Boschi[4], Fabio Cammarano[1]

[1]Dipartimento di Scienze, Università degli Studi Roma Tre, Rome, Italy
[2]Research School of Earth Sciences, The Australian National University, Canberra, Australia
[3]Istituto Nazionale di Geofisica e Vulcanologia, Sezione di Bologna, Bologna, Italy
[4]Dipartimento di Geoscienze, Università degli Studi di Padova, Padua, Italy

1 Resolution test

The ability of the data to resolve the lateral differences was assessed by performing a resolution or checkerboard test. Surface-wave propagation times are calculated, at each relevant period, based on a theoretical model where phase-velocity varies according to the corresponding inset in fig. S1, with a peak-to-peak velocity anomaly of 20% (from -10% to +10%). In the inversion, parameterization cells are 0.2º in lateral extent. The input model is most successfully recovered near the center of the area of interest, where ray path coverage is maximum. Smearing is seen, as expected, near its outer borders, where coverage is relatively poor.

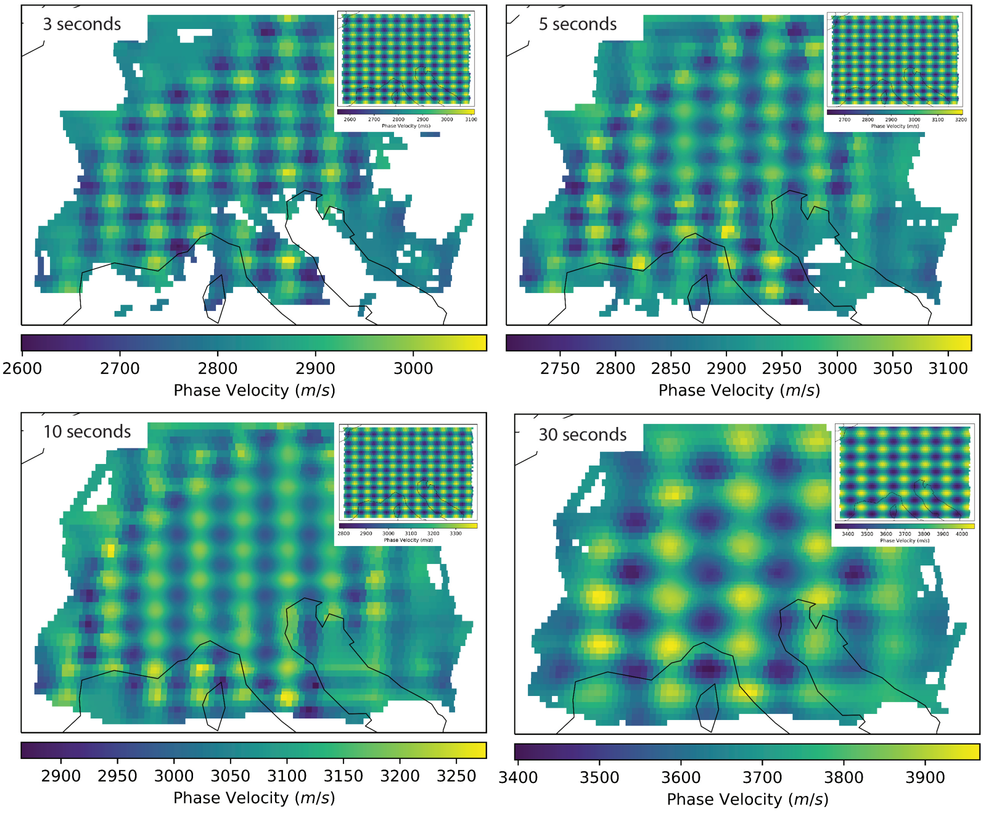

Figure S1. Checkerboard test at 3, 5, 10, and 30 seconds. The insets show the input models.

2 Test on the amount and distribution of stations pairs per subarray

To better assess the limitations of the method applied in this study, a test to understand the effect of the number of stations inside each subarray to the final value of attenuation coefficient was performed. A specific subarray located in a complex setting, i.e. between the Adriatic Sea, the Alps, the Dinarides and the Po Plain, with 39 stations and 368 cross-correlations was analyzed. Randomly eliminating stations and inverting for α each time showed us that the effect of fewer stations, azimuthally distributed in a different way, is particularly small on the final attenuation values (Fig. S2).

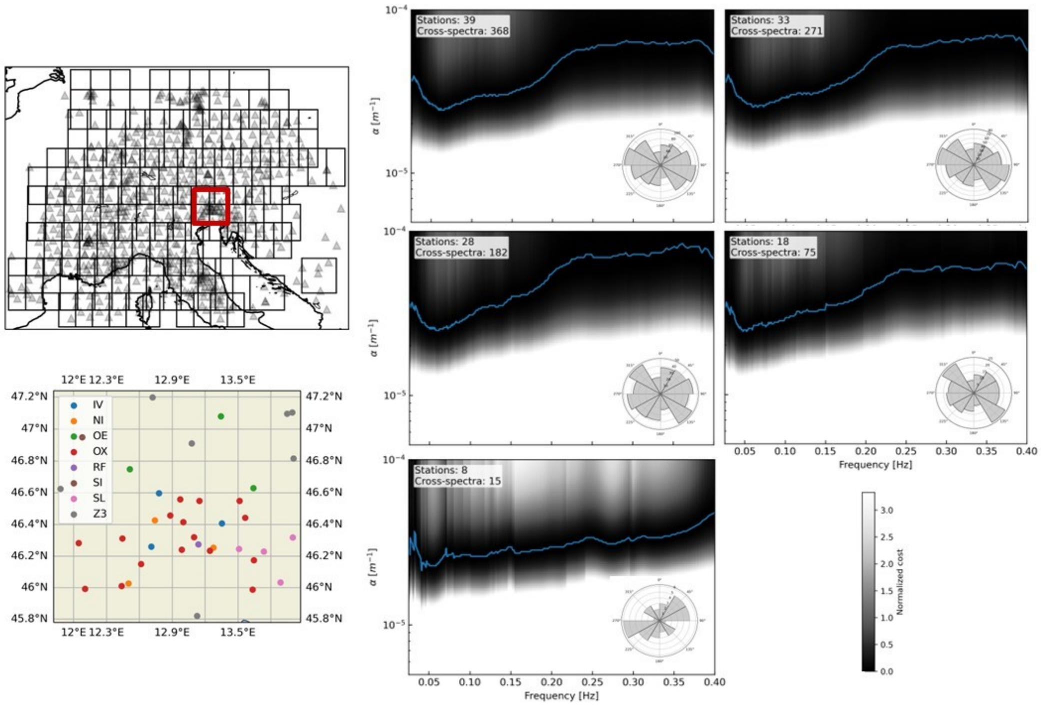

Figure S2. Top left panel: map with seismic stations used in our regional study. The subarrays are the black squares that have the same size as the red highlighted square, which is the subarray used for our test. Bottom left panel: this subarray presents 39 broadband stations from different networks. Right panels: sequentially eliminating stations (from 39 down to 8 stations) from this subarray shows that the effect on the final α value is small. The polar plot inside each panel represent the distribution of the remaining seismic stations.
